# Measurement of Serum Parameters Attributes the Therapeutic Effects of Music Therapy to Augmented Stress‐Coping Ability and Diminished Systemic Inflammation

**DOI:** 10.1155/da/4189470

**Published:** 2026-06-30

**Authors:** Yuchen Lu, Junyuan Yao, Ke-sheng Gu, Anbiao Wu, Xiqin Yang, Hao Wu, Lijun Xiao, Jiyan Zhang

**Affiliations:** ^1^ Department of Molecular Immunology, Beijing Institute of Basic Medical Sciences, Beijing, China, bjmu.edu.cn; ^2^ Department of Medical Psychology, The Third Medical Center of Chinese PLA General Hospital, Beijing, China, 301hospital.com.cn; ^3^ Department of Psychiatry, No. 984 Hospital of the PLA, Beijing, China; ^4^ Integrated TCM and Western Medicine Department, No. 984 Hospital of the PLA, Beijing, China; ^5^ Department of Medical Psychology, The Ninth Medical Center of Chinese PLA General Hospital, Beijing, China; ^6^ Chinese Institute for Brain Research, Beijing, China

**Keywords:** inflammation, MDD, music therapy, serum marker, stress

## Abstract

Major depressive disorder (MDD) is a leading cause of disability worldwide. It remains elusive whether serum levels of cortisol, amino acids and their downstream neurotransmitters, and inflammatory cytokines can reflect the therapeutic response of MDD. Although music seems to be beneficial to MDD treatment, the optimal treatment strategy remains to be explored. Here, we report that the combination of pharmacotherapy and music therapy with both D major K. 448 and G minor K. 550, but not pharmacotherapy alone, significantly downregulated or presented numerical trends to downregulate serum levels of cortisol and norepinephrine (NE) in MDD patients. Furthermore, serum levels of L_Histidine, L_Glutamic acid, L_Aspartic acid, tumor necrosis factor‐α (TNF‐α), TNF‐receptor I (TNFR I), and C–C motif ligand 11 (CCL11) were significantly downregulated or exhibited numerical trends to be downregulated by the combination of pharmacotherapy and music therapy, whereas those of cluster of differentiation 30 (CD30) exhibited opposite effects. Therefore, music therapy with both D major K. 448 and G minor K. 550 should be recommended as adjuvant therapy to MDD patients. Certain serum markers can reflect the therapeutic response of MDD. The therapeutic effects of music therapy may be attributed to augmented stress‐coping ability and diminished systemic inflammation.

**Summary**

• Music therapy with both D major K. 448 and G minor K. 550 exhibits beneficial effects on MDD patients.
• Pharmacotherapy alone and the combination of pharmacotherapy and music therapy exert distinct effects on serum marker levels.
• The therapeutic effects of music therapy are associated with augmented stress‐coping ability and diminished systemic inflammation.

## 1. Introduction

Major depressive disorder (MDD) is a leading cause of disability worldwide [1]. Treatment resistance and relapse are common [2]. However, it remains difficult to evaluate the treatment response [1]. Stress is associated with MDD and can trigger the release of hormone glucocorticoids (in humans, mainly cortisol) into the bloodstream via the hypothalamic–pituitary‐adrenocortical (HPA) axis [3, 4]. Although meta‐analytic studies have confirmed the correlation of HPA axis activity with MDD, there is considerable variability across studies [5]. Some amino acids can cross the blood‐brain barrier (BBB) and are converted to neurotransmitters. They have been regarded as potential biomarkers for depression [6]. Meanwhile, numerous studies indicate the association of peripheral inflammatory markers with MDD [7–10]. Whether these markers can reflect the treatment response remains to be clarified.

The combination of pharmacotherapy and other treatment procedures might be promising [2]. Music therapy is a potentially effective nonpharmaceutical treatment for MDD patients [11, 12]. Music has been shown to alleviate stress‐induced activation of the HPA axis via modulating oxidative stress and inflammation in adult mice [13, 14]. Accordingly, Mozart’s symphony Number 40 in G minor K. 550 has been reported to possess strong effects on lowering the subjects’ blood pressure, heart rate, and serum cortisol levels [15]. The suppression of stress‐induced oxidative stress and neuroinflammation by music also protects neurons from loss and maintains synaptic integrity [13, 14]. Furthermore, pleasant music has been demonstrated to stimulate the dopaminergic reward pathway and the mirror neuron system involved in empathy and learning [16–18]. Indeed, it is reported that Mozart’s Sonata for two pianos in D major K. 448 has the “Mozart effect,” which has been proven to enhance brain capacity and improve emotion [19, 20]. Even though high‐intensity music therapy seems to have better effects than low‐intensity [12], the optimal treatment strategy remains to be explored.

Here, we report that the combination of pharmacotherapy and music therapy with both D major K. 448 and G minor K. 550, but not pharmacotherapy alone, led to consistent changes of serum parameters in MDD patients. The therapeutic effects of music therapy are associated with augmented stress‐coping ability and diminished systemic inflammation.

## 2. Materials and Methods

### 2.1. Subjects and Study Design

This work has been carried out in accordance with the Helsinki Declaration of 1975, as revised in 2024. A total of 48 subjects were selected for this study, including 41 hospitalized MDD patients and 7 healthy controls (HCs). To better explore the effects of pressure, fireman patients were selected with a depression scale. Questionnaires included the Hamilton Anxiety Scale (HAMA), Hamilton Depression Scale (HAMD), Generalized Anxiety Disorder (GAD‐7), and Patient Health Questionnaires (PHQ‐9). Patients with any score greater than 8 were hospitalized. The inclusion criteria for HC samples included (1) sex and age matching with depression patients. (2) In good physical and mental health with no history of a mental illness. (3) A PHQ‐9 questionnaire score of less than 5.

We first included two cohorts of MDD patients and 7 HCs. One cohort (*n* = 17; the drug cohort) took antidepressants during hospitalization, whereas the other cohort (*n* = 15; the music cohort) listened to Mozart music in the morning from Monday to Friday while taking antidepressants. The music therapy employed Mozart’s Sonata for two pianos in D major K. 448 and Mozart’s symphony Number 40 in G minor K. 550. The total time was about 45 min. Two psychiatric nurses were responsible for escorting patients to and from the music therapy room, a 20 m^2^ room with soundproof panels on the walls. The room temperature was kept at around 25°C. No electronic device or book was allowed into the room. Seated in a comfortable armchair, patients were suggested to close their eyes and concentrate on the music. A speaker was used with a volume of 60–75 db. The two nurses monitored the condition throughout the session. In either cohort, the treatment lasted for 3 weeks. To confirm the effects of music therapy, we next included another music cohort of 9 patients (named as music cohort 2). All subjects signed written consent.

### 2.2. Traditional Chinese Medicine (TCM) Scores

Prior to formal assessment, physicians are required to independently conduct the four fundamental diagnostic procedures of TCM—inspection, listening and smelling, inquiry, and palpation—to establish a preliminary judgment regarding the nature and location of the disorder. Subsequently, to evaluate each item on the syndrome scoring scale—including primary symptoms, secondary symptoms, and associated signs (such as “fatigue and lack of strength,” “aversion to cold with cold limbs,” and “pale tongue with white coating and deep‐thin pulse”), clinicians must integrate patient‐reported information (inquiry), direct observations (inspection and listening/smelling), and findings from pulse and palpation diagnosis. According to the scale’s criteria (none, mild, moderate, relatively severe, and severe), symptom severity should be graded while simultaneously considering key TCM diagnostic dimensions such as yin–yang, exterior–interior, deficiency–excess, and cold–heat. This comprehensive assessment aims to accurately reflect the patient’s true symptom presentation related to depressive disorders over the past 1–2 weeks.

### 2.3. Sample Collection

Blood samples were collected from the subjects between 6:00 A.M. and 8:00 A.M. via venipuncture and were placed in SST tubes. The tubes were then centrifuged for 10 min at 3000 rpm and 25°C. After centrifugation, the serum was subsequently removed and stored in 1 mL aliquots at −80°C until further analysis.

### 2.4. Analysis of Serum Cortisol

The levels of serum cortisol were measured with an ELISA kit (Cat# ab108665, Abcam) according to the manufacturer’s protocol.

### 2.5. Analysis of Serum Neurotransmitters

Samples were thawed on ice. 10 μL serum was mixed with 70 μL methanol solution containing an internal standard (Sigma) dissolved in ultrapure water, followed by oscillation at 1400 rpm for 20 min at 10°C. The samples were then centrifuged at 18,000 g for 20 min at 4°C. After vacuum drying, 50 μL phenol isothiocyanate derivatized solution was added and kept at 30°C for 30 min, followed by drying with nitrogen. Then, 200 μL of 5 mM ammonium acetate in methanol solution was added for resolubilization, shaking for 30 min, and centrifugation for 10 min, 50 μL of supernatant was taken into a new 96‐well plate, 50 μL of deionized water was added, and the mixture was prepared for sample injection analysis. An ultrahigh pressure liquid chromatography‐triple quadrupole mass spectrometer (UPLC‐TQ‐MS, ACQUITY‐I UPLC/Xevo TQ‐S) from Waters Corporation was used to detect neurotransmitter‐related metabolites. The ACQUITY UPLC BEH C18 1.7 µM analytical column (2.1 × 100 mm) was used with column temperature 45°C and sample manager temperature 10°C. For mobile phases, A = 0.1% formic acid water and B = 0.1% formic acid acetonitrile/methanol (95:5). Gradient conditions were set up as follows: 0–1 min (1%–15% B), 1–2 min (15%–19% B), 2–3 min (19% B), 3–4.9 min (19%–70% B), 4.9–5 min (70%–100% B), 5–5.8 min (100% B), 5.8–6 (100%–1% B), and 6–6.6 min (1% B). The flow rate was 0.4 mL/min. The injection volume was 0.5 μL. For the mass spectrometer, the capillary was 3 kV (ESI+) with a source temperature of 150°C and a desolvation temperature of 500°C. The desolvation gas flow was 1000 L/h.

The raw data generated by UPLC‐TQ‐MS were analyzed with Waters MassLynx software (v4.1, Waters, Milford, MA, USA) for peak extraction, integration, identification, and quantitative analysis of each metabolite. The analyte concentration (×) of an unknown sample was calculated by comparing the unknown to a set of standard samples of known concentration (i.e., calibration curve) with the equation *y* = *ax* + *b*, where *y* is the instrument response such as peak height or area, a represents the slope/sensitivity, and *b* is a constant that describes the background. The *R* ^2^ of each calibration curve is >0.99.

### 2.6. Analysis of Serum Cytokines

Custom‐made Luminex assays (LXSAHM, R&D Systems, Minneapolis, MN, USA) were used to detect serum levels of cytokines according to the manufacturer’s guidelines. Briefly, supernatants were 1:2 diluted and incubated with the microparticle cocktail on a shaker (800 rpm) for 2 h at 25°C. Then, microparticles were washed and incubated with the biotin‐antibody cocktail on a shaker (800 rpm) for 1 h at 25°C. Microparticles were washed again and incubated with streptavidin‐PE on a shaker (800 rpm) for 30 min at 25°C. Subsequently, microparticles were washed and diluted in wash buffer. The results were generated by a Bio‐Plex 200 analyzer in 90 min (Bio‐RAD, Hercules, CA, USA). The cytokine concentration (pg/mL) of an unknown sample was calculated by comparing the unknown to a set of standard samples of known concentration (i.e., calibration curve) with the equation *y* = a + ([b–a]/[1 + ((x/c)^d^)]^f^), where a is the minimum asymptote, *b* is the maximum asymptote, *c* is the concentration at 50% of maximum effect, *d* is the hill slope, and *f* is the asymmetry factor. The *R* ^2^ of each calibration curve is >0.99.

### 2.7. Statistical Analysis

Error bars are shown as mean ± SD, and quantitative data were analyzed using Prism 6.0 (GraphPad). A two‐tailed Student’s *t*‐test, a one‐way ANOVA, and a two‐way ANOVA were used to evaluate quantitative variables that passed the normality test (Shapiro–Wilk test) and the homogeneity of variance test (Brown–Forsythe test). The Wilcoxon rank‐sum test was used to evaluate quantitative variables that failed to pass the Shapiro–Wilk test. Kruskal–Wallis was used to evaluate quantitative variables that failed to pass the Brown–Forsythe test. *p* < 0.05 was considered significant. 0.05 ≤ *p* < 0.15 was regarded as a numerical trend.

## 3. Results

### 3.1. Clinical Data Analysis of the Drug Cohort and the Music Cohort

**Figure 1:**
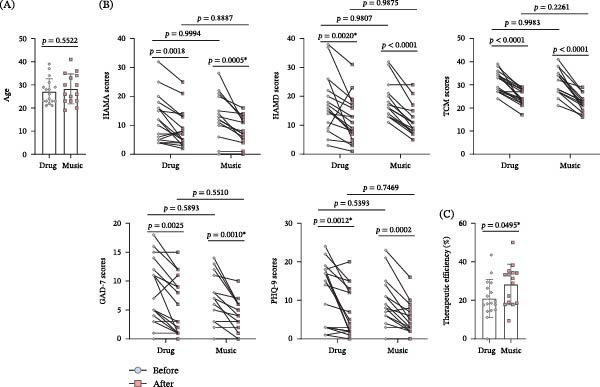
Clinical data analysis of the drug cohort and the music cohort. (A) Ages of participants in the drug cohort (Drug, *n* = 17) and the music cohort (Music, *n* = 15). Error bars show mean ± SD. The *p* value is shown above with a two‐tailed unpaired Student’s *t*‐test. (B) Hamilton Anxiety Scale (HAMA), Hamilton Depression Scale (HAMD), Generalized Anxiety Disorder‐7 (GAD‐7), Patient Health Questionnaire‐9 (PHQ‐9), and traditional Chinese medicine (TCM) scores before and after the treatment. Two‐way ANOVA was employed for the intergroup comparison between the drug cohort and the music cohort. A two‐tailed paired Student’s *t*‐test or paired Wilcoxon rank‐sum test was employed for the intragroup comparison before and after the treatment. *p* Values are shown above. (C) The therapeutic efficiency was calculated as (TCM score before the treatment − TCM score after the treatment) × 100 (%)/TCM score before the treatment. Error bars show mean ± SD. The *p* value is shown above with unpaired Wilcoxon rank‐sum test. Please note that Wilcoxon rank‐sum test (indicated with the symbol “ ^∗^”) was used to evaluate quantitative variables that failed to pass the normality test.

To better explore the effects of pressure, two cohorts of fireman MDD patients were first recruited. The recruitment commenced in June 2024 and was completed in September 2024. One cohort (*n* = 17; the drug cohort) took antidepressants during hospitalization, whereas the other cohort (*n* = 15; the music cohort) listened to Mozart music in the morning from Monday to Friday while taking antidepressants. There was no significant difference in terms of age between the two cohorts (Figure 1A). Meanwhile, there were also no significant differences in terms of HAMA, HAMD, GAD‐7, PHQ‐9, and TCM scores between the drug cohort and the music cohort before the treatment (Figure 1B). The music therapy employed Mozart’s Sonata for two pianos in D major K. 448 and Mozart’s symphony Number 40 in G minor K. 550. The total time was about 45 min. In either cohort, the treatment lasted for 3 weeks. According to these questionnaires, both treatment procedures showed good therapeutic effects. HAMA, HAMD, GAD‐7, and PHQ‐9 scores were comparable between the two cohorts after treatment (Figure 1B). Despite that, the *p* value in terms of TCM scores between the drug cohort and the music cohort after the treatment was 0.2261 (Figure 1B). In this scenario, we calculated the therapeutic efficiency according to the TCM scores. As expected, the combination of pharmacotherapy and music therapy (in brief, the music therapy) showed higher therapeutic efficiency than pharmacotherapy alone (i.e., antidepressants) (Figure 1C, *p* = 0.0495).

### 3.2. Changes of Serum Cortisol and Amino Acids in the Drug Cohort and the Music Cohort

**Figure 2:**
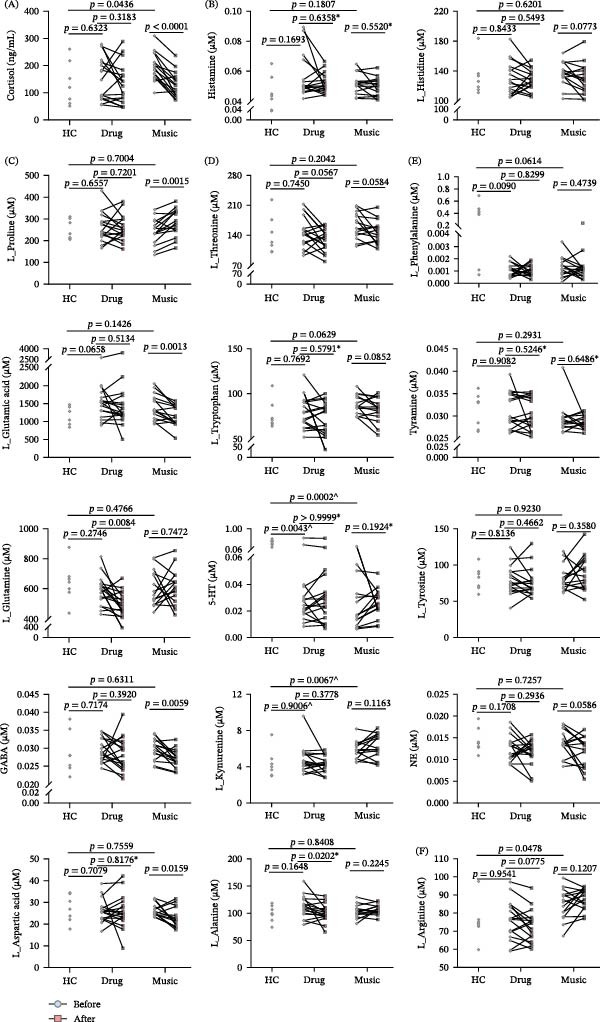
Changes of serum cortisol and amino acids in the drug cohort and the music cohort. (A) Cortisol. (B) Histamine and L_Histidine. (C) L_Proline, L_Glutamic acid, L_Glutamine, gamma‐aminobutyric acid (GABA), and L_Aspartic acid. (D) L_Threonine, L_Tryptophan, 5‐hydroxytryptophan (5‐HT), L_Kynurenine, and L_Alanine. (E) L_Phenylalanine, tyramine, L_Tyrosine, and norepinephrine (NE). (F) L_Arginine. One‐way ANOVA or Kruskal–Wallis was employed for the comparison among healthy controls (HC, *n* = 7), the drug cohort (Drug, *n* = 17), and the music cohort (Music, *n* = 15) before the treatment. A two‐tailed paired Student’s *t*‐test or paired Wilcoxon rank‐sum test was employed for the intragroup comparison before and after the treatment. *p* Values are shown above. Please note that Kruskal–Wallis (indicated with the symbol “^”) and Wilcoxon rank‐sum test (indicated with the symbol “ ^∗^”) were used to evaluate quantitative variables that failed to pass the homogeneity of variance test and normality test, respectively.

In this scenario, we also recruited 7 age‐matched male HCs to analyze serum markers. Serum cortisol levels correlate with MDD with considerable variability [5]. In this study, the music cohort, but not the drug cohort, showed higher levels of serum cortisol than HCs before the treatment (Figure 2A). Accordingly, the music therapy led to a significant reduction of serum cortisol levels, whereas the role of antidepressants is not obvious (Figure 2A).

As a precursor to histamine, L_Histidine can get into the brain via specific transporters, whereas histamine barely penetrates the BBB. Serum levels of L_Histidine have been reported to be reduced in female MDD patients [21, 22] but exhibit no significant changes in cohorts containing males [23, 24]. Compared to HCs, the two cohorts in our study did not show significant changes in serum levels of histamine and L_Histidine before the treatment (Figure 2B). The two treatment procedures exerted no significant effect on serum histamine levels (Figure 2B). Serum L_Histidine levels were not affected by antidepressants either but displayed a numerical trend to be lower after the music therapy (Figure 2B).

L_Glutamate (L_Glutamic acid) is an important excitatory neurotransmitter in the CNS and a precursor to L_Glutamine, gamma‐aminobutyric acid (GABA), and mitochondrial TCA cycle intermediates. L_Aspartate (L_Aspartic acid) is also an important excitatory neurotransmitter in the CNS and might share the same receptor with L_Glutamic acid [25]. Both L_Glutamic acid and L_Aspartic acid can cross the BBB via specific transporters [26]. Several studies have demonstrated the elevation of L_Glutamic acid levels in the blood of MDD patients [23, 27–29], whereas serum levels of its upstream L_Proline [23] and its downstream L_Glutamine [27, 29] seem to remain largely unchanged. Serum levels of GABA were reported to be downregulated or remain unchanged in MDD patients [29, 30]. Serum L_Aspartic acid levels were reported to be upregulated or remain unchanged in MDD patients [23, 27, 29]. As compared to HCs, the two cohorts displayed numerical trends of elevated serum levels of L_Glutamic acid and unchanged serum levels of L_Proline, GABA, L_Glutamine, and L_Aspartic acid before the treatment (Figure 2C). The music therapy significantly downregulated serum levels of L_Glutamic acid, GABA, and L_Aspartic acid, upregulated those of L_Proline, but showed no effect on those of L_Glutamine (Figure 2C). On the other hand, antidepressants significantly downregulated serum levels of L_Glutamine but failed to alter those of the other four parameters (Figure 2C).

L_Tryptophan is an essential amino acid that is the sole precursor of serotonin (i.e., 5‐hydroxytryptophan, 5‐HT), and it can also be catabolized into L_Kynurenine [22]. L_Tryptophan and L_Kynurenine can cross the BBB via specific transporters, whereas 5‐HT cannot [26]. Through the kynurenine pathway, L_Tryptophan is also metabolized into L‐alanine [31]. In addition, L_Threonine might affect the conversion of L_Tryptophan [32]. Previous observations have shown that there are no consistent elevation or reduction of these metabolites in the blood of MDD patients [22, 23, 27, 29]. Our two cohorts showed comparable serum levels of L_Threonine and L‐alanine to HCs before the treatment. The two treatment procedures exhibited numerical trends to reduce serum levels of L_Threonine. Antidepressants, but not the music treatment, also significantly lowered those of L‐alanine (Figure 2D). The drug cohort also displayed comparable serum levels of L_Tryptophan and L_Kynurenine to HCs before the treatment, and antidepressants failed to affect these two parameters (Figure 2D). Meanwhile, the music cohort presented higher serum L_Kynurenine levels than HCs with a numerical trend of higher serum L_Tryptophan levels before the treatment. The treatment procedure exhibited numerical trends to reverse the aberrance of L_Tryptophan and L_Kynurenine (Figure 2D). In addition, the two cohorts all showed elevated levels of serum 5‐HT before the treatment, as compared to HCs, which were not affected by either antidepressants or the music therapy (Figure 2D). Thus, the elevation of serum 5‐HT may be influenced by diet‐ and/or hospitalization‐related factors.

L_Phenylalanine is an important precursor of L_Tyrosine [22, 23]. L_Tyrosine can cross the BBB and serve as the precursor to norepinephrine (NE). NE is a neurotransmitter. It is also released by the sympathetic nervous system [33]. Tyramine can be generated through the decarboxylation of L_Tyrosine [34]. In line with previous observations that there are no consistent changes of these metabolites in the blood of MDD patients [22, 23, 27, 29], the two cohorts in our study displayed comparable levels of serum L_Tyrosine, tyramine, and NE to HCs before the treatment (Figure 2E). Despite that, music therapy presented a numerical trend to lower serum NE levels (Figure 2E). Notably, the drug cohort exhibited lower levels of serum L_Phenylalanine than HCs, and the music cohort displayed a similar numerical trend before the treatment, which were not affected by either treatment (Figure 2E).

L_Arginine is a precursor to nitric oxide, a signaling molecule in the brain. The music cohort, but not the drug cohort, presented higher serum L_Arginine levels before the treatment, in line with previous reports [23, 24]. The two treatment procedures showed numerical trends to lower serum L_Arginine levels (Figure 2F).

### 3.3. Alterations of Serum Cytokines in the Drug Cohort and the Music Cohort

**Figure 3:**
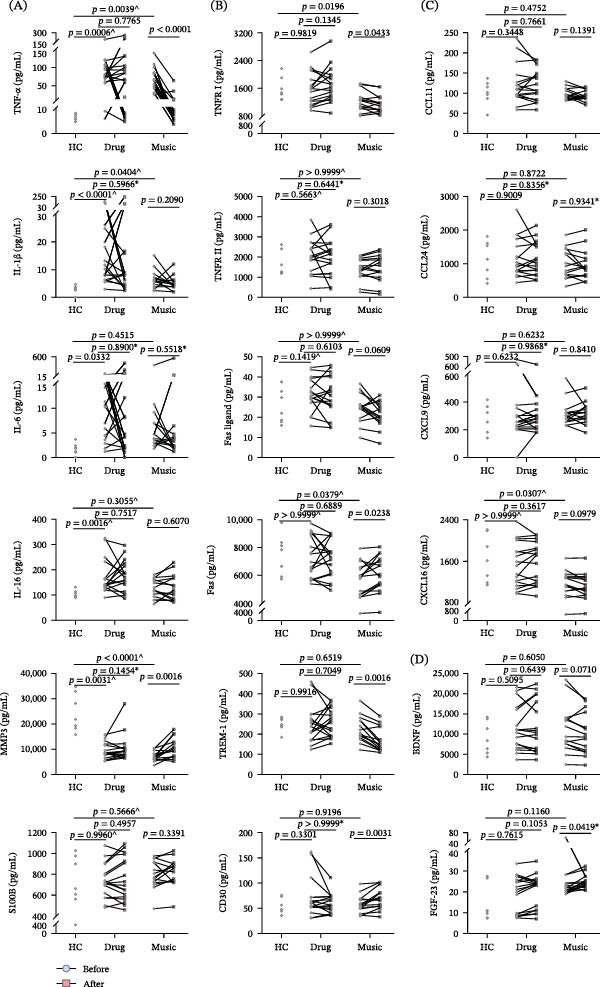
Alterations of serum cytokines in the drug cohort and the music cohort. (A) Proinflammatory cytokines: tumor necrosis factor‐α (TNF‐α), interleukin‐1β (IL‐1β), interleukin‐6 (IL‐6), interleukin‐16 (IL‐16), matrix metalloproteinase 3 (MMP3), and S100 calcium‐binding protein B (S100B). (B) Parameters, which are usually membrane‐bound molecules TNF‐receptor I (TNFR I), TNF‐receptor II (TNFR II), Fas ligand, Fas, triggering receptor expressed on myeloid cells‐1 (TREM‐1), and cluster of differentiation 30 (CD30). (C) Chemokines C–C motif ligand 11 (CCL11), C–C motif ligand 24 (CCL24), C–X–C motif ligand 9 (CXCL9), and C–X–C motif ligand 16 (CXCL16). (D) Growth factors: brain‐derived neurotrophic factor (BDNF) and fibroblast growth factor‐23 (FGF‐23). One‐way ANOVA or Kruskal–Wallis was employed for the comparison among healthy controls (HCs, *n* = 7), the drug cohort (Drug, *n* = 17), and the music cohort (Music, *n* = 15). A two‐tailed paired Student’s *t*‐test or paired Wilcoxon rank‐sum test was employed for the intragroup comparison before and after the treatment. *p* Values are shown above. Please note that Kruskal–Wallis (indicated with the symbol “^”) and Wilcoxon rank‐sum test (indicated with the symbol “ ^∗^”) were used to evaluate quantitative variables that failed to pass the homogeneity of variance test and normality test, respectively.

A meta‐analysis of 107 studies with 5166 MDD patients and 5083 controls indicates peripheral elevation of tumor necrosis factor‐α (TNF‐α) and interleukin‐6 (IL‐6) [35], whereas measuring interleukin‐1β (IL‐1β) poses a challenge due to its low concentration, even with high‐sensitivity assays [36]. Meanwhile, it is reported that some MDD patients with high serum C‐reactive protein (CRP) levels show elevated serum interleukin‐16 (IL‐16) levels [37]. The two cohorts in our study displayed higher serum TNF‐α levels than those of HCs before the treatment (Figure 3A). The music therapy led to a significant reduction of serum TNF‐α levels, whereas antidepressant treatment exerted no effects (Figure 3A). The two cohorts also exhibited higher serum IL‐1β levels than those of HCs before the treatment (Figure 3A). However, either treatment failed to change serum IL‐1β levels (Figure 3A). Moreover, some individuals in the drug cohort exhibited elevated serum levels of IL‐6 and IL‐16 before the treatment, but either treatment failed to alter these two parameters (Figure 3A). Matrix metalloproteinases (MMPs) play a key role in inflammation, and an association between MMP3 and depressive disorder has been reported [8]. In our hands, however, the two cohorts in our study displayed lower serum MMP3 levels than those of HCs before the treatment (Figure 3A). The music therapy led to significant elevation of serum MMP3 levels, and antidepressants presented a numerical trend (Figure 3A). Serum levels of S100 calcium‐binding protein B (S100B), produced primarily by astrocytes, are reported to be higher or remain unchanged in MDD patients [38, 39]. Compared to HCs, the two cohorts in our study did not show significant changes in serum levels of S100B before the treatment, and the two treatment procedures exhibited no effects (Figure 3A).

We also analyzed some serum markers, which are usually membrane‐bound molecules. TNF‐α signals through binding to TNF‐receptor I (TNFR I) and II (TNFR II) [40]. A meta‐analysis has reported higher serum levels of TNFR I and TNFR II in severe mental illness, including MDD [40]. Fas has been associated with depressive symptoms of dementia [41]. As a recently identified pattern recognition receptor, triggering receptor expressed on myeloid cells‐1 (TREM‐1) triggers the secretion of proinflammatory mediators from phagocytes but has not been implicated in MDD [42]. Similarly, no association between serum cluster of differentiation 30 (CD30) levels and moderate‐severe depression was found [43]. The drug cohort displayed unchanged serum levels of TNFR I, TNFR II, Fas, TREM‐1, and CD30 with a numerical trend of higher serum levels of the Fas ligand before the treatment (Figure 3B). Antidepressants exhibited a numerical trend to upregulate serum levels of TNFR I without affecting the other five parameters (Figure 3B). The music cohort showed lower serum levels of TNFR I and Fas before the treatment (Figure 3B). However, the music therapy lowered serum TNFR I and TREM‐1 levels and upregulated serum Fas and CD30 levels. The music therapy also presented a numerical trend to downregulate serum Fas ligand levels but failed to affect serum TNFR II levels (Figure 3B).

About chemokines, platelet factors C–C motif ligand 11/24/26 (CCL11/24/26) and C–X–C motif chemokine receptor 6 (CXCR6) ligands C–X–C motif ligand 9/16 (CXCL9/16) have been reported to be peripheral markers of damaged cognition [44, 45]. The two cohorts in our study did not exhibit significant peripheral upregulation of these chemokines before the treatment (Figure 3C). By contrast, the music cohort showed lower serum CXCL16 levels before treatment (Figure 3C). Antidepressants failed to affect these parameters. However, the music therapy displayed a numerical trend to downregulate serum levels of CCL11 and CXCL16 without affecting serum levels of CCL24 and CXCL9 (Figure 3C).

About growth factors, it has been reported that MDD patients with remitted and persistent depression, but not incident depression, show lower serum brain‐derived neurotrophic factor (BDNF) levels as compared to HCs [46]. Women with postpartum depressive symptoms have been reported to present higher serum fibroblast growth factor‐23 (FGF‐23) levels [47]. The two cohorts in our study exhibited similar serum BDNF levels to that of HCs before the treatment (Figure 3D). The drug cohort also showed similar serum FGF‐23 levels to HCs, but the music cohort exhibited a numerical trend of elevated serum FGF‐23 levels (Figure 3D). Antidepressants displayed a numerical trend to upregulate serum FGF‐23 levels without affecting serum BDNF levels. The music therapy upregulated serum FGF‐23 levels with a numerical trend to lower serum BDNF levels (Figure 3D).

### 3.4. Partial Consistent Changes of Serum Cortisol and Amino Acids in Another Cohort With Music Therapy

**Figure 4:**
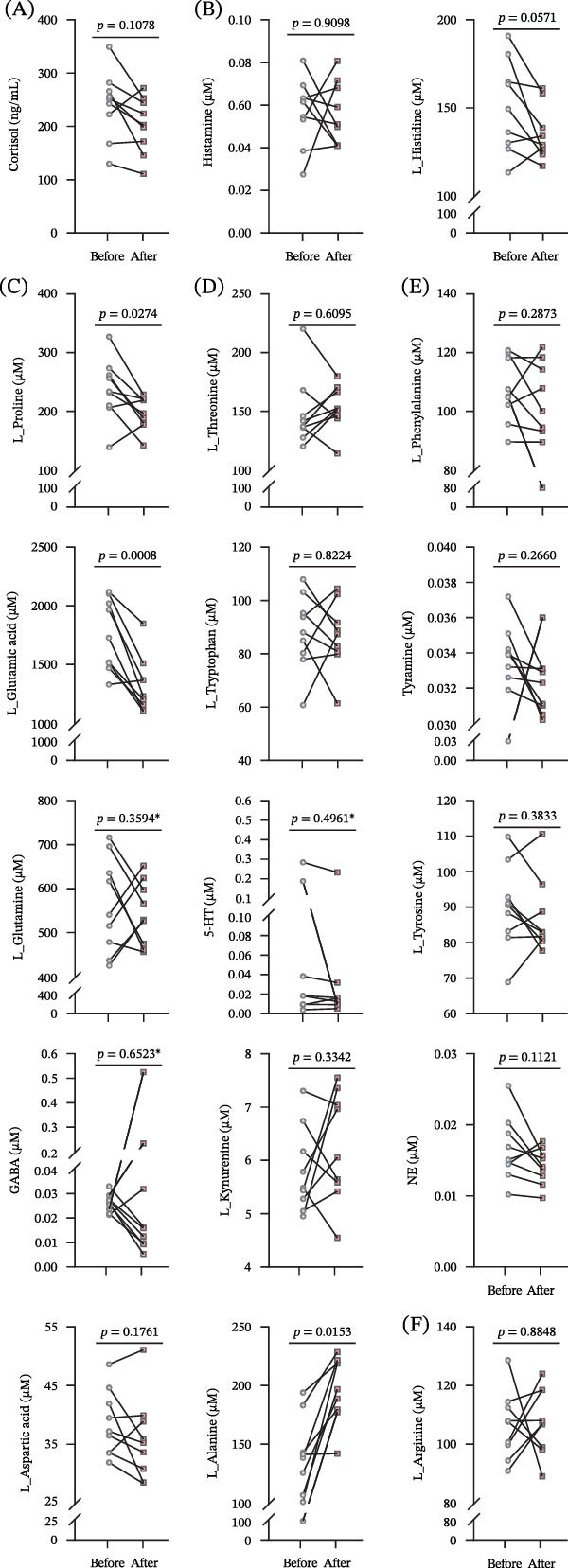
Partial consistent changes of serum cortisol and amino acids in another cohort with music therapy. (A) Cortisol. (B) Histamine and L_Histidine. (C) L_Proline, L_Glutamic acid, L_Glutamine, gamma‐aminobutyric acid (GABA), and L_Aspartic acid. (D) L_Threonine, L_Tryptophan, 5‐hydroxytryptophan (5‐HT), L_Kynurenine, and L_Alanine. (E) L_Phenylalanine, tyramine, L_Tyrosine, and norepinephrine (NE). (F) L_Arginine. A two‐tailed paired Student’s *t*‐test or paired Wilcoxon rank‐sum test was employed for the intragroup comparison before and after the treatment of the music cohort 2 (*n* = 9). *p* Values are shown above. Please note that the Wilcoxon rank‐sum test (indicated with the symbol “ ^∗^”) was used to evaluate quantitative variables that failed to pass the normality test.

To replicate the results above, we used another independent sample set that consisted of 9 fireman MDD patients who agreed to undergo the music therapy (the music cohort 2). In line with the aforementioned data, the music therapy displayed numerical trends to lower serum levels of cortisol (Figure 4A) and L_Histidine (Figure 4B) and significantly downregulated serum levels of L_Glutamic acid (Figure 4C) in music cohort 2. The effects of the music therapy on serum levels of L_Aspartic acid (Figure 4C) and NE (Figure 4E) in music cohort 2 were weaker than expected. Moreover, the effects of the music therapy on serum levels of GABA (Figure 2C), L_Threonine, and L_Tryptophan (Figure 2D) in the music cohort disappeared in the music cohort 2 (Figure 4D). In contrast to the upregulation of serum L‐proline levels in the music cohort after the treatment (Figure 2C), the downregulation of serum L‐proline levels in the music cohort 2 was observed after the treatment (Figure 4C). In addition, the music therapy showed no effects on serum L‐alanine levels in the music cohort (Figure 2D) but significantly upregulated serum L‐alanine levels in the music cohort 2 (Figure 4D).

### 3.5. Partial Consistent Changes of Serum Cytokines in Another Cohort With Music Therapy

**Figure 5:**
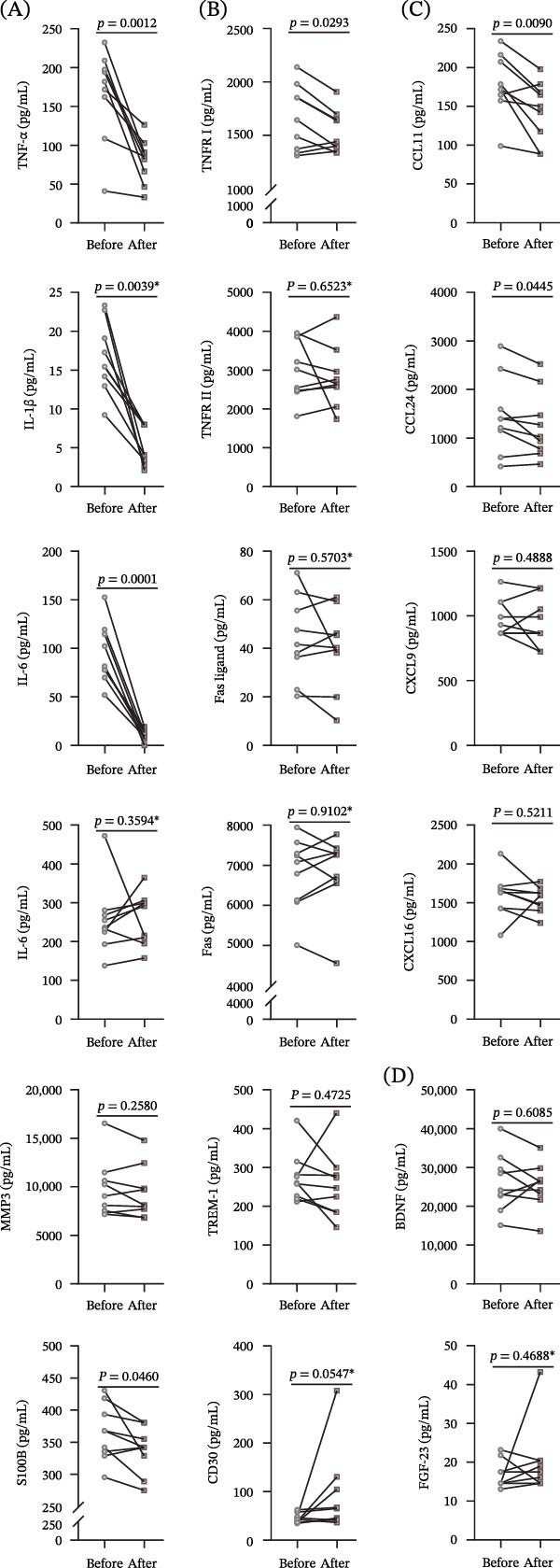
Partial consistent changes of serum cytokines in another cohort with music therapy. (A) Proinflammatory cytokines: tumor necrosis factor‐α (TNF‐α), interleukin‐1β (IL‐1β), interleukin‐6 (IL‐6), interleukin‐16 (IL‐16), matrix metalloproteinase 3 (MMP3), and S100 calcium‐binding protein B (S100B). (B) Parameters, which are usually membrane‐bound molecules TNF‐receptor I (TNFR I), TNF‐receptor II (TNFR II), Fas ligand, Fas, triggering receptor expressed on myeloid cells‐1 (TREM‐1), and cluster of differentiation 30 (CD30). (C) Chemokines C–C motif ligand 11 (CCL11), C–C motif ligand 24 (CCL24), C–X–C motif ligand 9 (CXCL9), and C–X–C motif ligand 16 (CXCL16). (D) Growth factors: brain‐derived neurotrophic factor (BDNF) and fibroblast growth factor‐23 (FGF‐23). Two‐tailed paired Student’s *t*‐test or paired Wilcoxon rank‐sum test was employed for the intragroup comparison before and after the treatment of the music cohort 2 (*n* = 9). *p* Values are shown above. Please note that the Wilcoxon rank‐sum test (indicated with the symbol “ ^∗^”) was used to evaluate quantitative variables that failed to pass the normality test.

We also checked the changes of serum cytokines in the music cohort 2 after the treatment. In line with the aforementioned data, the music therapy significantly downregulated serum levels of TNF‐α (Figure 5A), TNFR I (Figure 5B), and CCL11 (Figure 5C) with a numerical trend to upregulate those of CD30 (Figure 5B) in the music cohort 2. Additionally, the music therapy significantly lowered serum levels of IL‐1β, IL‐6, S100B (Figure 5A), and CCL24 (Figure 5C) in music cohort 2. However, the effects of the music therapy on serum levels of MMP3 (Figures 3A and 5A), Fas ligand, Fas, TREM‐1 (Figures 3B and 5B), CXCL16 (Figures 3C and 5C), BDNF, and FGF‐23 (Figures 3D and 5D) in the music cohort disappeared in the music cohort 2.

## 4. Discussion

Our data indicate that the combination of pharmacotherapy and music therapy possesses better therapeutic effects than pharmacotherapy alone according to TCM scores. It is not surprising that HAMA, HAMD, GAD‐7, and PHQ‐9 scores did not accurately reflect the therapeutic effects. Our study suggests that serum markers can be used for this purpose. After 3 weeks of treatment, antidepressants even displayed a numerical trend to upregulate serum levels of TNFR I, which is associated with depressive symptoms [35]. Furthermore, antidepressants significantly lowered serum levels of L_Glutamine. As dietary L_Glutamine can alleviate mood disorders [48], the downregulation of its serum levels might reinforce emotional defects. It is known that antidepressants might worsen depressive symptoms during the first 2 weeks. The aggravating effects of antidepressants might result from the changes in these parameters, which can last up to 3 weeks. However, the combination of pharmacotherapy and music therapy for 3 weeks lowered or demonstrated numerical trends to lower serum levels of TNF‐α, TNFR I, and cognition injury marker CCL11 [44] while exerting opposite effects on those of CD30 in two independent cohorts. Meanwhile, the music therapy could maintain serum levels of L_Glutamine. These data suggest that the inclusion of music therapy might strengthen the therapeutic effects of antidepressants. More importantly, music therapy seems to be of potential clinical significance in preventing the initial aggravating effects of antidepressants. Notably, although the music cohort 2 provides some supportive evidence for serum parameters, the replication was only partial. These facts reflect individual differences and the complexity of MDD.

Mozart’s Sonata for two pianos in D major K. 448 has long been associated with the “Mozart effect” [19, 20], possibly because it stimulates the dopaminergic reward pathway and the mirror neuron system involved in empathy and learning [16–18]. Our data about the cognition injury marker CCL11 in two independent music therapy cohorts may reflect the “Mozart effect.” The HPA axis helps the body control the response to stress by releasing cortisol [49]. Stress induces oxidative stress and inflammation to over‐activate the HPA axis, which can be reversed by music in adult mice [13, 14]. As stated above, the therapeutic effects of music therapy are associated with diminished systemic inflammation in our patients with music therapy. Accordingly, while listening to symphony Number 40 in G minor K. 550, one of the greatest works of Wolfgang Amadeus Mozart, has been reported to lower serum cortisol levels [15]. It was written in 1788, a dark year for Mozart with bills piling up and an infant daughter dying. This might be the reason that this symphony contains unusual anxiety. However, Mozart never allowed his music to stay long in sadness. While spurring tears, this symphony also provokes delight. Maybe, such a transition fits the feelings of MDD patients better. Moreover, our patients might gain higher empathy ability after listening to D major, K. 448. Our data about serum levels of cortisol and NE after combined pharmacotherapy and music therapy are consistent with the literature, suggesting an augmented stress‐coping ability. Therefore, sequential administration of D major K. 448 and G minor K. 550 in each workday morning may be superior to other reported music therapy schedules for MDD patients, especially those with cognitive injury.

According to previous findings in adult mice [13, 14], diminished systemic inflammation in our patients with the music therapy should contribute to augmented stress‐coping ability. Meanwhile, the changes of serum cytokines after music therapy might also result from an augmented stress‐coping ability. Even though both glucocorticoids and NE directly inhibit the expression of proinflammatory cytokines in monocytes, their persistent elevation in the blood causes monocyte expansion and mobilization from the bone marrow [50–52]. Augmented stress‐coping ability might alleviate such aberrations. Serum CD30 levels are associated with augmented Th2 activity [53]. TNF‐α signaling has been reported to inhibit Th2 activity [54]. Thus, the elevation of serum CD30 levels after the music therapy might result from reduced serum TNF‐α levels. Similarly, the changes of L_Glutamic acid and L_Histidine after the music therapy might also be the consequences of augmented stress‐coping ability. Local NE and L_Glutamic acid release reciprocally enhance the release of each other [55]. On the other hand, glucocorticoids can downregulate histidine decarboxylase [56] and thereby upregulate serum L_Histidine levels. Heart rate variability (HRV) has been recognized as a psychological stress indicator, and disrupted sleep quality is believed to link stress to depression [57, 58]. Thus, monitoring HRV and sleep quality as well as stress assessment questionnaires can further test these possibilities in future work.

## Funding

This study is supported by grants from the National Natural Science Foundation of China (Grants 82530063, 81930027, and 92169207 to Jiyan Zhang).

## Ethics Statement

The study was approved by the Ethics Committee of the Beijing Institute of Basic Medical Sciences (AF/SC‐08/02.430) and the Ethics Committee of PLA General Hospital (KY2023‐002). All subjects signed written consent for the treatment and/or for using samples and data in research and publication.

## Conflicts of Interest

The authors declare no conflicts of interest.

## Data Availability

The data that support the findings of this study are available from the corresponding authors upon reasonable request.
